# Factor XI Inhibitors: perspectives in primary and secondary prevention of ischemic stroke

**DOI:** 10.1007/s11739-024-03611-w

**Published:** 2024-05-14

**Authors:** Domenico Prisco, Maria Canfora, Matteo Mazzetti, Irene Mattioli, Alessandra Bettiol

**Affiliations:** 1 Department of Experimental and Clinical Medicine, University of Florence, Viale L.Go Giovanni Brambilla, 3, 50134 Florence, Italy; 2 Internal Interdisciplinary Medicine Unit, Careggi University Hospital, Florence, Italy

**Keywords:** Anticoagulants, Atrial fibrillation, Factor XI inhibitors, Stroke

## Abstract

Stroke is one of the most common causes of mortality and disability worldwide. Antithrombotic therapy represents the mainstay in primary and secondary prevention, both in cardioembolic and non-cardioembolic stroke. Particularly, direct oral anticoagulants play a crucial role in atrial fibrillation, the most common cause of cardioembolic stroke, whereas single or dual antiplatelet therapy is preferred in non-cardioembolic stroke. However, the limitations related to the residual risk of cardioembolic or cerebrovascular events, and the risk of major bleeding, still represent unmet medical needs. To overcome them, new drugs inhibiting Factor XI (FXI) and Factor XII have been proposed, with a selective inhibition of contact pathway of coagulation, delineating a new anticoagulant approach. This review provides a summary of the currently available evidence and future perspectives on FXI inhibitors, that can represent an additional therapeutic option in the primary and secondary prevention of cardioembolic and non-cardioembolic ischemic stroke, also in challenging therapeutic contexts.

**Graphical Abstract:**

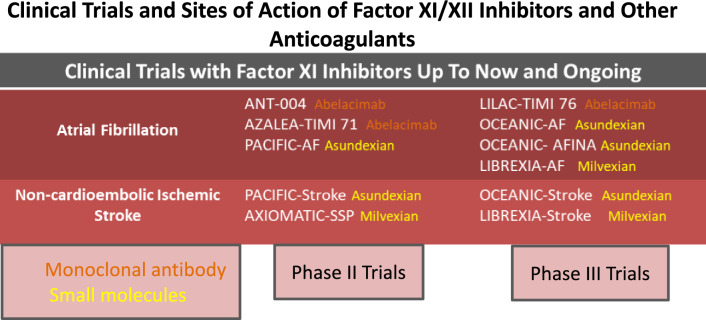

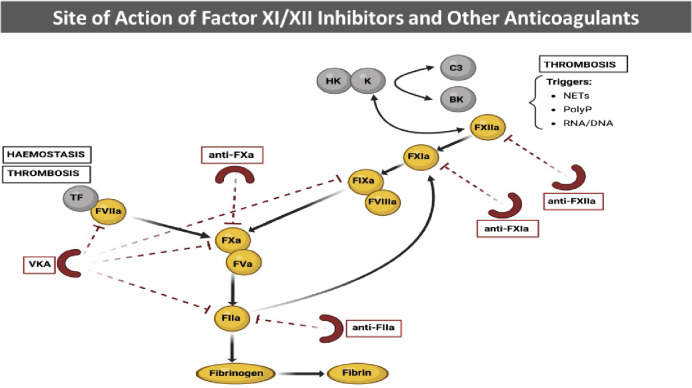

## Introduction

Ischemic stroke is the second most common cause of mortality and the third leading cause of combined mortality and disability globally [1]. Given the high risk of recurrence, determining the etiology of an ischemic stroke is mandatory to set up an effective secondary prevention. Up to 75% of ischemic strokes can be attributed to non-cardioembolic origin, whereas the remaining 25% have a cardioembolic etiology, already known or detected during the diagnostic work-up [2].

Atrial fibrillation (AF) is the most common cause of cardioembolic stroke, due to the formation of blood clots in the left atrial appendage, and it necessitates anticoagulation therapy if not otherwise contraindicated. Over the past 10 years, direct oral anticoagulants (DOACs) have become the mainstay for the prevention of stroke and systemic embolism (SE) in patients with non-valvular atrial fibrillation (NVAF). DOACs, which target either thrombin (FIIa) or activated coagulation Factor X (FXa), are now preferred over vitamin K antagonist (VKA) for the main indications, thanks to their non-inferior efficacy, with a better safety profile and a more convenient therapeutic management. In NVAF, DOACs have been associated with a significant reduction in ischemic stroke or systemic embolic events (19%), hemorrhagic stroke (51%) and all-cause mortality (10%) compared with VKAs. However, they were also associated with a 25% greater risk of gastrointestinal bleeding, with the possible exception of apixaban [3]. The use of DOACs in NVAF is still burdened by a residual risk of embolic events and, on the other side, by an increased residual risk of intra- and extracranial bleeding [3–6]. Conversely, single or dual antiplatelet therapy (SAPT or DAPT) is preferred in non-cardioembolic ischemic stroke, where endothelial injury or arterial plaque disruption might be involved, to reduce the risk of recurrences. DAPT, taken for a limited time in the acute phase of high-risk transient ischemic attack (TIA) or non-cardioembolic minor ischemic stroke, appears to provide more benefits than SAPT, particularly in the first 21 days after the onset of symptoms [7–9]. Particularly, in the POINT trial, patients with minor ischemic stroke and high-risk TIA had a risk of ischemic stroke within 90 days of 6.3% when treated with SAPT (i.e., aspirin alone) and 4.6% when treated with DAPT (i.e., the combination of aspirin and clopidogrel), with an increase in major bleeding [8]. Indeed, the risk of bleeding continues to represent an important issue especially when antithrombotic drugs are administered.

To overcome these limitations, both in the context of cardioembolic and non-cardioembolic stroke, new generation anticoagulants are emerging in clinical research as promising alternatives. In recent years, driven by the search for an anticoagulant as effective as DOACs but with a more favorable safety profile, drugs inhibiting Factor XI (FXI) or Factor XII (FXII) have been proposed [10], to selectively inhibit the contact pathway of coagulation, with the final aim of saving physiological hemostasis with prevalent inhibition of thrombogenesis, thus dissociating for the first time these two mechanisms [11, 12].

**Fig. 1:**
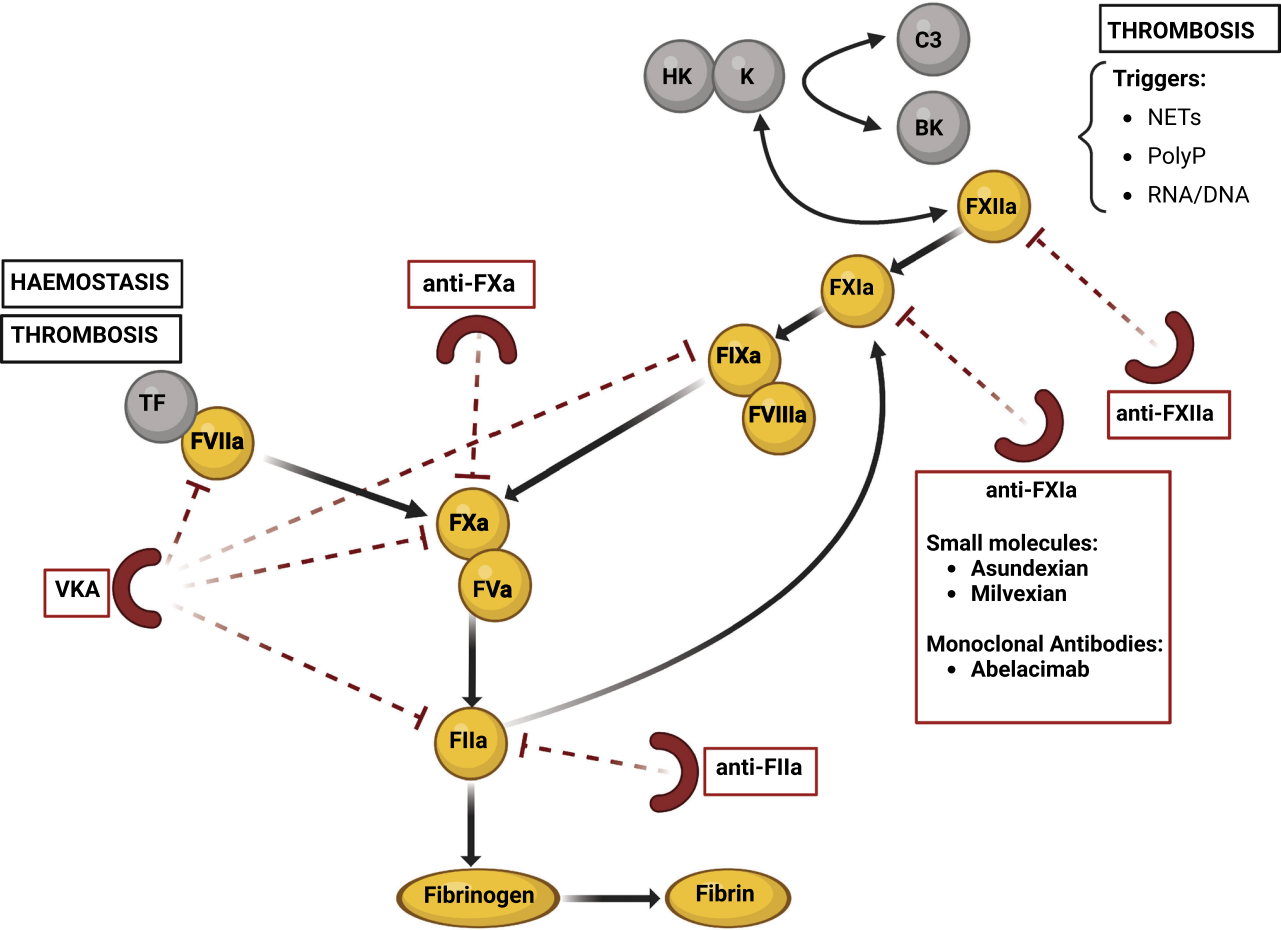
Clotting pathway and sites of action of different anticoagulants. *VKA* vitamin K antagonist, *TF* tissue factor, *HK* high molecular weight kininogen, *K* Kallikrein, *BK* Bradykinin,* NETs* neutrophil extracellular traps, *PolyP* patogen-associated polyphosphates, *C3* complement component 3

FXI and FXII are two circulating glycoproteins involved in the contact pathway of coagulation (Fig. 1). They circulate in the blood as inactive zymogens that can be (auto)activated to FXIa and FXIIa in response to extracellular nucleic acids, neutrophilic extracellular traps, pathogen-associated polyphosphates, or negatively charged surfaces coming in contact with the blood [10]. All these triggers, when present in a growing thrombus, can activate FXII which subsequently activates FXI. Emerging data show that FXI has a minor role in the physiological process of hemostasis but an important role in the development of thrombosis [11–13].

Actually, in vivo hemostasis depends primarily on the extrinsic pathway (tissue factor) of coagulation, with the intrinsic pathway (including FXI) providing amplification of this process with a role only subsidiary in preventing bleeding. On the other side, FXI has a predominant role in thrombus growth and progression, thanks to its ability to generate thrombin [12–14], eventually leading to vessel occlusion and pathological manifestations of thrombosis. In the thrombotic process, FXIIa is considered the primary activator of FXI, in particular when blood is exposed to artificial surfaces such as mechanical heart valves or medical devices, to polyphosphates released by activated platelets or to other activators such as nucleic acids or fragments of atherosclerotic plaques [11–14]. Also, FXIIa activates prekallikrein, stimulating in turn further FXII activation via a positive feedback mechanism.

The great interest in FXI and FXII as potential therapeutic targets for safe and effective antithrombotic strategies comes from the knowledge gained through the study of natural models of the rare hereditary deficiencies of FXI (also called hemophilia C) and FXII. Congenital FXI deficiency is a rare condition (prevalence of homozygous mutation 1/100000) with a higher frequency (8–9%) in some groups including Ashkenazi Jews and Iraqi Jews [15]. Unlike the X-linked FVIII deficiency (haemophilia A) or FIX deficiency (haemophilia B), the FXI deficiency is rarely associated with spontaneous bleeding; hemorrhagic episodes can occur after surgical procedures or trauma in areas with high fibrinolytic activity [16–18]. On the other hand, in this population there is a significant reduction of arterial and venous thrombosis compared to the general population [19]. Particularly, inherited FXI deficiency has been associated with a significantly reduced incidence of ischemic stroke [20], but not of myocardial infarction [20, 21]. More recently, novel evidence that a genetic predisposition to lower FXI levels is associated to less frequent ischemic stroke and VTE events, without an increased risk of major bleeding has been reported. Data from 371,695 participants in the UK Biobank and two large genome-wide association studies [22] showed that genetically lower FXI levels were associated with lower risk of ischemic stroke [odds ratio (OR) 0.47, 95% confidence interval (CI) 0.36–0.61; *p* < 0.001] and venous thromboembolism (OR 0.1, 0.07–0.14; *p* < 0.001), but not with major bleeding (OR = 0.7, 0.45–1.04) [22]. Of note, from the available information on ischemic stroke subtypes, a strong risk reduction was found for the cardioembolic stroke subtype (OR 0.16, 0.09–0.28; *p* < 0.001) [22]. Moreover, a follow-up analysis in the UK Biobank showed that, amongst individuals with atrial fibrillation, those with genetically lower levels of factor XI were at reduced risk of all ischemic stroke, compared to those with normal levels of factor XI. The hazard ratios (HR) for the association between genetically lowered FXI levels with ischaemic stroke were 1.44 (95% CI 0.90–2.31) in individuals with atrial fibrillation and 1.10 (1.02–1.19) in controls without atrial fibrillation [23]. These findings strengthen the role of components of the intrinsic pathway of coagulation, as FXI, in the pathogenesis of cardioembolic and non-cardioembolic stroke.

As FXII is concerned, studies conducted in patients with moderate to severe FXII deficiency did not show an increase in the risk of bleeding nor a reduction in thrombotic risk, but FXII up-regulation has been associated with increased mortality [24–26]. Indeed, FXII is essential for the propagation and growth of a developing thrombus, especially device-induced, but not for hemostasis [27].

To date, FXII inhibitors are mostly undergoing preclinical or phase I clinical investigation, whereas several phase II and phase III studies with FXI inhibitors have been conducted or are currently ongoing.

The aim of this review is to summarize current evidence and future perspectives regarding FXI inhibitors in the context of primary and secondary prevention of cardioembolic and non-cardioembolic stroke.

## FXI inhibitors for stroke prevention in atrial fibrillation

Based on their chemical structure, several categories of FXI inhibitors have been developed up to now, including monoclonal antibodies, antisense oligonucleotides (ASO), small molecules, aptamers or natural inhibitors, and some of them have reached phase II and phase III of clinical development (Table 1).

**Table 1:** Phase II or III clinical trials with factor XI inhibitors in AF and IS or TIA

Drug	Structure	Phase	Trial	Population	Number	Comparator	Status
Abelacimab	Fully human monoclonal antibody	IIa	ANT-004 (NCT04213807)	AF	28	Placebo	Completed
		IIb	AZALEA TIMI 71 (NCT04755283)		1200	Rivaroxaban	Stopped early
		III	LILAC-TIMI 76 (NCT05712200)		1900	Placebo	Ongoing
Asundexian	Small molecule	II	PACIFIC-AF (NCT04218266)	AF	753	Apixaban	Published
		III	OCEANIC-AF (NCT05643573)		18,000	Apixaban	Stopped early
		III	OCEANIC-AFINA		2000	Placebo	Planned; not recruiting
Milvexian	Small molecule	III	LIBREXIA-AF (NCT05757869)	AF	15,500	Apixaban	Ongoing
Asundexian	Small molecule	IIb	PACIFIC-Stroke (NCT04304508)	IS or TIA	1808	Placebo on top to SAPT or DAPT	Published
		III	OCEANIC-Stroke (NCT05686070)		9300	Placebo	Ongoing
Milvexian	Small molecule	II	AXIOMATIC-SSP (NCT03766581)	IS or TIA	2366	Placebo on top to SAPT or DAPT	Published
		III	LIBREXIA Stroke (NCT05702034)		15,000	Placebo on top to SAPT or DAPT	Ongoing

*AF* atrial fibrillation, *IS* ischemic stroke, *TIA* transient ischemic attack, *SAPT* single antiplatelet therapy, *DAPT* dual antiplatelet therapy

Regarding monoclonal antibodies, the first phase I and phase II trials were conducted with abelacimab (MAA868). Abelacimab is a fully human monoclonal antibody that binds to the catalytic domain of FXI, blocking it in the conformation of an inactive zymogen [28, 29].

Abelacimab, which has an intravenous or subcutaneous administration route, has a fast onset of action (peak plasma concentration of 1.75–2 h) and a relatively long half-life (25–30 days), allowing monthly administration. Furthermore, it should be noted that monoclonal antibodies such as abelacimab lack renal clearance and are not removed by dialysis, which differentiates them from small molecules [10, 28].

In a first-in-human phase I study, single subcutaneous administration of abelacimab up to the 240 mg dose was found to be safe and well-tolerated leading to dose dependent reduction in free FXI levels and prolongation of aPTT with abelacimab doses 150 mg and higher [29]. A phase I study of single ascending doses of intravenous abelacimab (30–150 mg) or placebo in healthy volunteers and healthy obese people has been recently completed (ANT-003) [29].

In the multiple ascending doses phase IIa ANT-004 trial (NCT04213807), abelacimab (120 or 180 mg) or placebo was subcutaneously administered once monthly for 3 months in patients with NVAF or flutter at low cardioembolic risk (CHA_2_DS_2_-VASc score 0–1 in male and 1–2 in female) in whom the use of an anticoagulant for stroke prevention was not suitable, according to the investigator’s judgment. Twenty-eight participants were screened; 5 participants did not meet inclusion/exclusion criteria; 5 eligible participants failed randomization. The primary outcome was the number of participants that achieved more than or equal to 50, 80, and 90% FXI inhibition after the third administration (Day 91) of the two different doses of abelacimab. From preliminary results, this monoclonal antibody appears to effectively inhibit more of 50% of FXI activity in the larger part of patients randomized to the 180 mg dose, leading to reductions in free FXI levels associated with rapid and sustained prolongation of the aPTT (Table 2) [29]. Furthermore, the ANT-003 and ANT-004 studies revealed that no patient developed a confirmed, treatment-emergent antidrug antibodies (ADA) response.

**Table 2:** Main results from published studies or preliminary data for stroke prevention in atrial fibrillation

Trial	Study drugs/comparator	Main results	
		Efficacy data	Safety data
ANT-004 (NCT04213807)	Abelacimab (120 or 180 mg sc once monthly for 3 months) or placebo	FXI inhibition ≥ 50%: 33.3 and 57.1% in abelacimab 120 and 180 mg, 0% in placebo. FXI inhibition ≥ 80%: 0 and 14% in abelacimab 120 and 180 mg, 0% in placebo. FXI inhibition ≥ 90%: 0% in all groups	All-cause mortality and serious adverse events: 0% in all groups
AZALEA-TIMI 71 (NCT04755283)	Abelacimab (150 or 90 mg sc monthly) or Rivaroxaban (20 mg daily orally*)	The composite rate of stroke and SE: 1.1 and 1.4 per 100 pt-years in abelacimab 150 and 90 mg respectively and 1.0 per 100 pt-years in the rivaroxaban group	The composite rate of major bleeding or CRNMB events (ISTH criteria): 2.7 and 1.9 per 100 pt-years in abelacimab 150 and 90 mg respectively; 8.1 per 100 pt-years in the rivaroxaban group
LILAC-TIMI 76 (NCT05712200)	Abelacimab (150 mg sc monthly) or placebo	The composite rate of ischemic stroke and SE: still ongoing	The composite rate of BARC 3c/5 bleeding: still ongoing
PACIFIC-AF (NCT04218266)	Asundexian (20 or 50 mg once daily) or apixaban (5 mg twice daily)	The composite rate of cardiovascular death, myocardial infarction, ischemic stroke or SE: 0.8 and 1.6% in asundexian 20 and 50 mg respectively, 1.2% in the apixaban 5 mg group	The composite rate of major bleeding and CRNMB (ISTH criteria): 1.2 and 0.4% in asundexian 20 and 50 mg respectively, 2.4% in the apixaban 5 mg group. The annualized IR (per 100 pt-years): 5.47 per 100 pt-years for asundexian 20 mg, not calculable for asundexian 50 mg, 11.10 per 100 pt-years for apixaban 5 mg
OCEANIC-AF (NCT05643573)	Asundexian (50 mg once daily) or apixaban (5 mg twice daily)	The composite rate of ischemic stroke and SE: trial prematurely closed. Data not yet published	The composite rate of major bleeding (ISTH criteria): trial prematurely closed. Data not yet published
LIBREXIA-AF (NCT05757869)	Milvexian (100 mg twice daily, orally) or apixaban (5 or 2.5 mg twice daily, orally)	The composite rate of stroke and non-CNS SE: still ongoing	The composite rate of major bleeding and CRNMB (ISTH criteria): still ongoing

*15 mg if CrCl ≤ 50 ml/min at randomization or during study

*CRNMB* clinically relevant non-major bleeding, *ISTH* International Society of Thrombosis and Hemostasis, *sc* subcutaneous, *SE* systemic embolism, *BARC* Bleeding Academic Research Consortium, *IR* incidence ratio, *Pt* patient, *CNS* central nervous system

The results of these clinical studies contributed to clarify the tolerability, safety, pharmacokinetic and pharmacodynamic of abelacimab, paving the way to the much larger AZALEA-TIMI 71 trial (NCT04755283).

The AZALEA-TIMI 71 is a phase IIb, multicenter, randomized, active-controlled study comparing two blinded doses of abelacimab (90 or 150 mg given by subcutaneous injection once monthly) with rivaroxaban 20 mg daily in patients with NVAF, who are at moderate to high risk for stroke, defined by age ≥ 55 years and CHA_2_DS_2_-VASc of ≥4 or a CHA_2_DS_2_-VASc of ≥3 in association with concomitant antiplatelet medication use or Creatinine Clearance (CrCl) ≤ 50 ml/min. The study enrolled 1287 patients from 95 centers in United States (U.S.), Canada, Europe and Asia. The median age was 74 years, 44% of patients were female and the median CHA_2_DS_2_-VASc score was 5; 92% of patients had already taken anticoagulants and 66% had taken DOACs. Patients were followed up for a median of 1.8 years [31]. In the overall trial cohort, ClCr < 50 ml/min was present in 21% of the participants; in patients with CrCl ≤ 50 ml/min at randomization or during the study, rivaroxaban 15 mg/die was used. The primary outcome was the effect of abelacimab compared to rivaroxaban on the rate of major bleeding or clinically relevant non-major (CRNM) bleeding as defined by International Society on Thrombosis and Haemostasis (ISTH) criteria.

In September 2023, Anthos Therapeutics announced that AZALEA-TIMI 71 study was stopped early due to an “overwhelming reduction” in bleeding with both the 150 and 90 mg doses [30]. Following the preliminary results reported at the *American Heart Association-Scientific Sessions 2023* on November 12th in Philadelphia, abelacimab demonstrated excellent anticoagulant activity with a median change in FXI from baseline to 3 months of −97% [interquartile range (IQR) −50 to −99] in the 90 mg group and −99% (IQR −98 to −99) in the 150 mg group. The composite rate of major and CRNM bleeding was 2.7 per 100 patient-years in patients taking abelacimab 150 mg (HR 0.33, 95% CI 0.19–0.55), 1.9 per 100 patient-years in the 90 mg abelacimab group (0.23, 0.13–0.42) and 8.1 per 100 patient-years in the rivaroxaban group (*p* < 0.001 for both doses of abelacimab as compared to rivaroxaban) with a reduction in the events of 67 and 77% respectively with abelacimab. Gastrointestinal (GI) bleeding occurred at a rate of only 0.1 per 100 patient-years with abelacimab at both doses, vs. 2.1 per 100 patient-years with rivaroxaban (HR 0.07, 95% CI 0.01–0.50 and 0.07, 0.01–0.51, respectively) indicating the almost total prevention of GI bleeding, frequently occurring in patients treated with DOACs and often leading to undertreatment. Major bleeding occurred at a rate of 1.0 per 100 patient-years in the 150 mg abelacimab group (HR 0.26, 95% CI 0.11–0.61) and 0.7 per 100 patient-years in the 90 mg abelacimab group (0.19, 0.07–0.50) vs. 3.7 events per 100 patient-years in the rivaroxaban group (*p* < 0.001 for both doses of abelacimab as compared to rivaroxaban). Similarly, CRNM bleeding occurred at rates of 1.8 per 100 patient-years (HR 0.39, 95% CI 0.21–0.75) and 1.1 per 100 patient-years (0.25, 0.11–0.54) in abelacimab 150 and 90 mg respectively, vs. 4.6 per 100 patient-years in the rivaroxaban group (*p* < 0.001 for both doses of abelacimab) (Table 2). Intracranial hemorrhage was uncommon and not significantly different between the groups. As secondary outcomes are concerned, such as the incidence of stroke and systemic embolism, ischemic or hemorrhagic stroke and all-cause death, no statistically significant differences were observed among the three treatment arms, but the trial was underpowered to evaluate an effect of the treatment on stroke and death (Table 2). Anyway, the rates of ischemic stroke were numerically but not significantly higher with abelacimab: 1.1 per 100 patient-years in the 150 mg group and 1.3 per 100 patient-years in the 90 mg group vs. 0.7 per 100 patient-years in the rivaroxaban group (*p* = 0.42 and 0.28, respectively). Considering a median CHA_2_DS_2_-VASc score of 5, one would expect an annual stroke rate of 6–7% without treatment [32], which suggests a comparable efficacy of DOACs and abelacimab in preventing around 70–80% of stroke events. As regards the net clinical outcome (a composite of ischemic stroke, systemic embolism, major or CRNM bleeding and all-cause death), statistically significant differences were found in favor of both abelacimab doses (150 and 90 mg) compared to rivaroxaban: 5.5 per 100 patient-years and 5.6 per 100 patient-years vs. 11.3 per 100 patient-years respectively (*p* < 0.001 for both comparisons).

In conclusion, the results of this phase II trial indicate that both doses of abelacimab (150 and 90 mg monthly) are superior to rivaroxaban 20 mg daily in reducing bleeding events among patients with NVAF and a high CHA_2_DS_2_-VASc score. For this reason, an extension study has started to enable patients to switch from rivaroxaban to abelacimab to benefit from the improved bleeding profile.

In the meantime, a phase III trial with abelacimab in AF patients is ongoing. The LILAC-TIMI 76 (NCT05712200) study is an event-driven, randomized trial aimed to evaluate the efficacy and safety of abelacimab as compared to placebo on the rate of ischemic stroke or systemic embolism and of BARC (Bleeding Academic Research Consortium) major bleeding (3c/5) in AF patients aged 65–74 years and with CHA_2_DS_2_-VASc ≥ 5, or age ≥ 75 years and with CHA_2_DS_2_-VASc ≥ 4, judged by the responsible physician or by their own decision to be unsuitable for currently available anticoagulation therapy. Patients are randomized to receive abelacimab 150 mg or matching placebo subcutaneously once monthly (Table 2). The researchers aim to enroll approximately 1900 patients from North America, Europe, Latin America, the Middle East, and Asia. This study is planned to end in March 2025.

Based on the available evidence, abelacimab appears to epitomize its promise as a hemostasis-sparing anticoagulant and could represent a paradigm shift in the prevention of stroke and other thrombotic conditions. Indeed, if an anti-FXIa therapeutic strategy will provide effective anticoagulation with a minor effect on hemostasis, we might be able to extend our ability to prevent unwanted thrombotic events to broader patient populations, including those at high risk for both bleeding and thrombosis who, because of their extreme fragility, often remain off anticoagulant treatment [33].

Beside monoloclonal antibodies, four anti-FXI small molecules are in advanced stages of clinical evaluation: asundexian, milvexian, SHR2285 and frunexian (EP-7041). The first three have a reversible effect and are administered orally; frunexian is administered intravenously.

To date, SHR2285 and frunexian have never been tested in patients with AF and non-cardioembolic ischemic stroke, so there are no data on their use in the clinical settings analyzed in the present review.

Regarding the small oral molecules asundexian and milvexian, thanks to their small size, rapid diffusion across the membranes is possible, with a rapid onset and offset of action (peak plasma concentration of 2–4 h, and fairly short half-life of 8–16 h), so as to require once or twice daily administration [10, 12, 13, 18, 34].

Asundexian was investigated in the PACIFIC-AF trial (NCT04218266), a multicenter, randomised, double-blind, phase II trial that compared two doses of asundexian (20–50 mg/day) with a standard regimen of apixaban (5 mg twice day, with dose reductions where necessary). The study was conducted at 93 sites in 14 countries, including 12 European countries, Canada, and Japan [35]. The aim of this study was to find the optimal dose of asundexian also comparing the incidence of bleeding with that of apixaban in patients with NVAF. In this study, 753 patients aged ≥45 years with NVAF, a CHA_2_DS_2_-VASc score of at least 2 if male or at least 3 if female, and an increased bleeding risk (history of previous bleeding requiring medical attention within 12 months, estimated glomerular filtration rate of 30–50 ml/min, or current indication for aspirin) were enrolled. Patients with another requirement for chronic anticoagulation, or on antiplatelet therapy (apart from up to 100 mg aspirin) were excluded from the study. Enrolled subjects had a mean age of 73.7 years; 351 (46%) were over 75 years, 309 (41%) were women, 336 (45%) were previously on DOACs; the mean CHA_2_DS_2_-VASc score was 4 (with 8.5% with a prior stroke). Overall, only 3% had a prior major bleeding event (slightly unbalanced, with up to 5.5% in the asundexian 50 mg group), and 28% had chronic kidney disease (slightly unbalanced, with only 21% in the asundexian 20 mg group).

After 4 weeks of treatment, asundexian 20 and 50 mg resulted in a 81 and 92% reduction in baseline FXIa at trough concentrations, and 90 and 94% at peak concentrations, respectively. After 12 weeks of therapy, both doses of asundexian were superior to apixaban in the primary safety outcome (major bleeding or CRNM bleeding according to ISTH criteria): three primary composite endpoint events occurred in the asundexian 20 mg arm, one in the asundexian 50 mg arm, and six in the apixaban arm. Overall, there were no episodes of ISTH major bleeding in any treatment arm. Ten patients experienced a CRNM bleeding event and 48 had any bleeding event. In general, bleeding rates were lower than expected during the planning of the study. The ratio of incidence proportions was 0.50 (90% CI 0.14–1.68) for asundexian 20 mg, 0.16 (CI 0.01–0.99) for asundexian 50 mg and 0.33 (0.09–0.97) for pooled asundexian vs. apixaban. The annualized incidence rate per 100 patient-years for the primary endpoint (major bleeding or CRNM bleeding) was 5.47 (90% CI 1.49–11.48) for asundexian 20 mg, not calculable for asundexian 50 mg, 3.61 (1.23–7.00) for asundexian total, 11.10 (4.83–19.45) for apixaban. Interestingly, these incidence rates found in the apixaban arm were similar to those of rivaroxaban in the AZALEA TIMI-71 trial, where the primary endpoint was the same. It is worth considering that this trial was designed as a dose-finding phase II clinical study, and was not powered to test differences in the rates of thrombotic events (Table 2). The rates of adverse events leading to discontinuation of the study drug were also similar in the three treatment arms.

Following the encouraging results of the PACIFIC-AF study, the phase III OCEANIC-AF study (NCT05643573) was started, aiming at evaluating the efficacy and safety (in terms of prevention of ischemic stroke, systemic embolism, and major bleeding) of asundexian (50 mg/day, orally) vs. apixaban (5 mg twice day) in adult patients with NVAF on a larger scale. The study was expected to enroll 18,000 patients aged ≥18 years and NVAF with CHA_2_DS_2_-VASc score ≥ 3 if male or ≥4 if female, or CHA_2_DS_2_-VASc score of 2 if male or 3 if female and enrichment criteria. This trial was expected to end in August 2025. However, on November 19th 2023, Bayer announced the early interruption of the OCEANIC-AF study: this decision by an independent data monitoring committee was based on ongoing surveillance findings showing that asundexian was inferior to apixaban for the prevention of stroke and systemic embolism in patients with AF (Table 2), marking the first unexpected negative result for this drug [36].

It is important to consider that the dosage chosen for the phase III trial was based on a phase II trial that was not powered to test differences in the rates of thrombotic events. It is therefore possible that the dosage chosen for the OCEANIC-AF trial was too low. Furthermore, when focusing on the early interruption of the OCEANIC-AF study with asundexian due to its low efficacy, as compared to the early interruption of the AZALEA-TIMI 71 trial with abelacimab for its superior safety with possible maintained efficacy, it could be speculated that a more intense and persistent inhibition of FXI occurs with abelacimab, due to its relatively long half-life, while oscillations over time in the extent of FXI inhibition might occur with small molecules, due to their shorter half-life. Certainly, the early termination of OCEANIC AF has raised many doubts and uncertainties for the future of asundexian in the field of atrial fibrillation.

Anyway, full data will further be analyzed and published to explain why asundexian did not meet the desired effect. OCEANIC-AFINA, a third randomized, placebo-controlled, double-blind phase III trial has been planned, in which asundexian should be compared with placebo in AF patients at high risk for stroke or systemic embolism who were deemed ineligible for oral anticoagulant therapy due to bleeding concerns. This trial has not yet started recruiting patients. Nevertheless, the study design will now be re-evaluated in light of the OCEANIC-AF interruption [36].

As for other small molecules, milvexian (BMS-986177/JNJ-70033093) is a direct-acting, high-affinity small molecule acting as inhibitor of FXIa. The LIBREXIA-AF (NCT05757869) is an ongoing phase III trial that evaluates the efficacy and safety of milvexian vs. apixaban in 15,500 patients from U.S, Canada, Australia, Europe, South Africa, with AF and CHA_2_DS_2_-VASc ≥ 2. The purpose of this study is to assess if milvexian is at least as effective as apixaban for reducing the risk of the composite stroke and non-central nervous system (CNS) systemic embolism (Table 2). The primary outcome will be the time to first occurrence of the composite endpoint of stroke and non-CNS systemic embolism, with up to 4 years of follow-up. This trial will end in May 2027.

In a recent metanalysis, Galli et al. [37] pooled the results coming from eight phase II randomized controlled trials comparing FXI inhibitors vs. other anticoagulants (enoxaparin or DOACs) or vs. placebo on top of antiplatelet therapy, including a total of 9216 patients. Two of these studies enrolling a total of 935 patients compared the FXI inhibitors with DOACs, and reported that FXI inhibitors were associated with a trend to reduce the endpoint “any bleeding” (RR 0.66, 95% CI 0.31–1.38) without any difference in major bleeding (1.03, 0.22–4.78) or in trial-defined efficacy endpoint (1.23, 0.88–1.70).

## FXI inhibitors for secondary prevention in non-cardioembolic stroke

The rate of early stroke recurrence after a non-cardioembolic ischemic stroke or transient ischemic attack (TIA) remains high. After a first ischemic event, the recurrence rate is estimated on average to be around 6%/year, despite the application of early therapeutic recommendations of current guidelines [38]. For this reason, FXI inhibitors represent a promising alternative also in this setting of secondary stroke prevention.

The PACIFIC-Stroke (NCT04304508) is a randomised, double-blind, placebo-controlled, phase IIb dose-finding trial, in which 1808 patients with acute (within 48 h) non-cardioembolic ischemic stroke (NIHSS ≤ 15) were randomized to asundexian (10, 20 or 50 mg/day, orally) or placebo, on top with SAPT or DAPT. Patients from 196 hospitals in 23 countries were enrolled if they were aged ≥45 years, to be treated with antiplatelet therapy, and able to have a baseline magnetic resonance imaging (MRI), either before or within 72 h of randomization [39].

At 26 weeks, the primary efficacy outcome of recurrent symptomatic ischemic stroke or covert brain infarcts detected by MRI at 6 months, occurred in 18.9, 22, 20.1 and 19.1% respectively in the three doses of asundexian and placebo; indeed, no dose response was observed. However, the analysis of secondary outcomes showed a reduction of the composite outcome of ischemic stroke or TIA with asundexian 50 mg compared to placebo (HR 0.64, 95% CI 0.41–0.98). Furthermore, an exploratory post-hoc subgroup analysis, showed that patients with any intra/extracranial atherosclerosis had fewer recurrent strokes and TIAs with asundexian 50 mg (HR 0.39, 90% CI 0.18–0.85). Major and/or CRNM bleeding (according to ISTH criteria) events were observed in 4.3, 3.1, 4.3, 2.4% of patients respectively on ascending dose of asundexian or placebo, without significant increase in bleeding and hemorrhagic transformation (Table 3). After the promising results of this trial, the ongoing phase III OCEANIC-Stroke (NCT05686070) has been planned, on 9300 patients aged ≥18 years, with an acute non-cardioembolic stroke or high-risk TIA and a systemic or cerebrovascular atherosclerosis or acute non-lacunar infarct. The main purpose of this study is to evaluate whether asundexian works better than placebo in reducing ischemic strokes when given in addition to standard antiplatelet therapy. Another aim is to compare the occurrence of major bleeding events during the study between the asundexian and the placebo group (Table 3). The results of this study are expected in October 2025.

**Table 3:** Main results from published studies or preliminary data for secondary prevention in non-cardioembolic stroke

Trial	Study drugs/comparator	Main results	
		Efficacy data	Safety data
PACIFIC-Stroke (NCT04304508)	Asundexian (10, 20 or 50 mg once daily) or placebo on top of SAPT or DAPT	The composite rate of symptomatic recurrent ischemic stroke and incident covert brain infarcts detected on follow-up MRI at 6 months: 18.9, 22, 20.1 and 19.1% in the asundexian 10, 20, 50 mg and in placebo group, respectively	The composite rate of major and/or CRNM bleeding (ISTH criteria): 4.3, 3.1, 4.3 and 2.4% in the asundexian 10, 20, 50 mg and in placebo groups, respectively
OCEANIC-Stroke (NCT05686070)	Asundexian (50 mg once daily) or placebo (on top of standard antiplatelet therapy)	Ischemic stroke: still ongoing	Major bleeding (ISTH criteria): still ongoing
AXIOMATIC-SSP (NCT03766581)	Milvexian (25 mg once daily, 25, 50, 100 or 200 mg twice) or placebo on top of DAPT (for 21 days) followed by ASA alone	The composite rate of new ischemic stroke or new covert brain infarction on MRI at 90 days: 16.7, 16.6, 15.6, 15.4, 15.3 and 16.8% in the milvexian 25 mg once daily, 25, 50, 100, 200 mg twice daily and in placebo groups, respectively	Event rate classified according to all BARC bleedings: 10.8, 8.6, 12.3, 13.1, 10.2 and 7.9% in the milvexian 25 mg once daily, 25, 50, 100, 200 mg twice daily and in placebo groups, respectively. Event rate classified according to BARC type 3 and 5 bleedings: 0.6, 0.6, 1.5, 1.6, 1.5, and 0.6% in 25 mg once daily, 25, 50, 100, 200 mg twice daily and in placebo groups, respectively. Also analyzed with ISTH definition
LIBREXIA Stroke (NCT05702034)	Milvexian 25 mg (twice a day, orally) or placebo (on top of SAPT or DAPT)	Ischemic stroke: still ongoing	Not provided

*ASA* acetyl-salicylic acid, *BARC* Bleeding Academic Research Consortium, *CRNMB* clinically relevant non-major bleeding, *DAPT* dual antiplatelet therapy, *ISTH* International Society of Thrombosis and Hemostasis, *MRI* magnetic resounance imaging, *SAPT* single antiplatelet therapy

The AXIOMATIC-SSP (NCT03766581) is an international, phase II, randomized, dose-ranging clinical trial examining the safety and efficacy of milvexian in patients with acute ischemic stroke (NIHSS ≤ 7) or high-risk TIA (ABCD^2^ score ≥ 6) and intra- or extracranial atherosclerotic plaque, enrolled within 48 h of the event. Overall, 2366 patients from 367 sites in 27 countries were randomized to milvexian (25 mg daily or 25, 50, 100 or 200 mg twice daily) or placebo, in combination with antiplatelet therapy (DAPT for 21 days followed by acetylsalicylic acid alone) [40]. The median age of enrolled patients was 71 years and 64% were males. The purpose of this trial was to determine whether the addition of an oral FXI inhibitor to aspirin and clopidogrel was more effective than the standard therapy in secondary stroke prevention. The results showed that the primary efficacy cumulative endpoint (symptomatic ischemic stroke at 90 days or an asymptomatic cerebral infarction on MRI) was not different in the milvexian and placebo group [41] (Table 3). However, data suggested a trend towards a lower risk of symptomatic ischemic stroke (excluding covert brain infarction) with milvexian (incidence rate of 4.6, 3.8, 4.0, 3.5% for ascending doses) as compared to placebo (5.5%), showing an approximately 30% relative risk reduction, except for the 200 mg twice daily dose (7.7%), for which no benefit was shown. The reasons for this unexpected finding have been object of debate. The main safety endpoint (major bleeding, defined as BARC type 3 and 5 bleeding) showed bleeding events between 0.6 and 1.6% without episodes of fatal bleeding; most of bleeding events were gastrointestinal. No dose response for major bleeding was established [40, 41].

Based on these results, the phase III LIBREXIA-Stroke Study (NCT05702034) is enrolling approximately 15,000 participants from Europe, Australia, Asia, South Africa, New Zealand, South America and U.S. aged ≥40 years who had experienced a non-lacunar acute ischemic stroke (NIHSS ≤ 7) or high-risk TIA (ABCD^2^ score ≥ 6) within 48 h, who received antiplatelet therapy as standard of care. The eligible patients were randomized to receive either milvexian or placebo twice a day orally within 48 h of their stroke or TIA, in addition to SAPT or DAPT. The primary outcome is the time to first occurrence of ischemic stroke (Table 3). This study will end in December 2026.

Notably, caution has been suggested on the role of high-dose FXI inhibitors when administered in addition to antiplatelet therapy, as reported by the results of the meta-analysis of three randomized controlled trials on a total of 5753 patients, performed by Galli et al. [37], comparing FXI inhibitors vs. placebo on top of SAPT or DAPT. The results showed that FXI inhibitors were associated with a 25% increase in the risk of any bleeding (RR 1.25, 95% CI 1.08–1.43), which was statistically significant for high dose (1.36, 1.09–1.70) but not for low dose of FXI inhibitors (1.17, 0.97–1. 42). No difference was reported in trial-defined efficacy endpoint (1.02, 0.92–1.13).

## Reversal strategies for FXI inhibitors

Although the risk of bleeding with FXI inhibitors is potentially low [37], a relevant point is the availability of strategies to reverse their pharmacological effect, to be used in case of bleeding. To date, specific antidotes are not currently available [42]. Tranexamic acid (1000–1500 mg or 15 mg/kg ev) appears to play a major role in the treatment of bleeding in patients receiving FXI inhibitors, considering the impairment of thrombin-activatable fibrinolysis inhibitor (TAFI) activation with FXI inhibition.

In case of life-threatening bleeding, reversal therapy with bypassing agents such as recombinant Factor VIIa (rFVIIa, 15–30 µg/kg ev) or activated prothrombin complex concentrate (aPCC, 50 UI/kg ev), if rFVIIa is not available, possibly associated with tranexamic acid, have been supposed. Other options are 3 or 4 factor prothrombin complex concentrate (PCC) or fresh frozen plasma (FFP) although evidence on this is currently limited [42].

However, one should consider that there was no need of reversal agents in the already completed phase II trials, which underscores that the need for reverse therapy with FXI inhibitors may be less relevant than with DOACs.

## FXI inhibitors in venous thromboembolism and other areas of unmet clinical needs

FXI inhibitors are potentially promising treatment strategies also in the setting of cancer-associated thrombosis (CAT). Indeed, this population is at high risk of both recurrences of thrombotic events and hemorrhages and is therefore a setting that could benefit from the potential mechanisms of these new molecules. Currently, two phase II trials are ongoing: the ASTER trial (NCT05171049) will randomize 1655 patients with non-skin cancer to abelacimab (150 mg intravenous administration, followed by monthly administration of the same dose subcutaneously) or apixaban (10 mg followed by 5 mg twice a day), whereas the MAGNOLIA trial (NCT05171075) will randomize 1020 patients with gastrointestinal or genitourinary cancer to abelacimab (same dose as in ASTER) or dalteparin (200 IU/kg/day followed by 150 IU/kg/day). Both trials will investigate VTE recurrence at 6 months and will use an open-label design with blinded assessment of the study outcomes. A small phase II trial assessing FXI inhibition with a monoclonal antibody, gruticibart (previously known as AB023), for the prevention of catheter-associated thrombosis in patients with cancer undergoing central line placement, has also been recently published [43]. Gruticibart was well tolerated without any significant adverse or bleeding related events and resulted in a lower incidence of thrombosis compared with the published literature [43].

Another area of potential interest for new anticoagulation strategy is antiphospholipid syndrome, where VKAs are recommended over DOACs, due to an increased risk of thromboembolic events with DOAC compared to VKA [44].

Furthermore, FXI inhibitors, due to their potential interest for the prevention of clotting induced by foreign surfaces, might be involved in future research in other areas of unmet clinical need, such as patients with mechanical heart valve or ECMO (Extracorporeal Membrane Oxygenation) [45].

Other critical settings for anticoagulation therapy include patients with end-stage renal disease or dialysis who are at high risk of both cardio- and cerebro-vascular events and bleeding tendencies. In this context, FXI inhibitors could play a major role: particularly, monoclonal antibodies and ASO are not cleared by kidney and are not removed by dialysis, which could result in favorable safety profiles in patients with renal impairment [18].

## Conclusions

The new frontier of anticoagulation, where bleeding may not necessarily represent the inevitable price to be paid, seems to belong to a very near future. Different phase II trials indicate an encouraging safety profile of the new FXI inhibitors in atrial fibrillation and non-cardioembolic ischemic stroke setting, with results in terms of efficacy yet to be defined. However, the premature termination of the OCEANIC-AF trial with asundexian represents a setback that leads to uncertainties regarding the effectiveness of these molecules, particularly in the prevention of cardioembolic stroke. The efficacy results of ongoing phase III studies will be necessary to better define the role of FXI inhibitors, which could represent a further therapeutic option not only for the prevention of ischemic stroke, redefining the relationship between efficacy and safety in these critical clinical scenarios.

## Data Availability

Not applicable.
